# The A Priori Traveling Repairman Problem

**DOI:** 10.1007/s00453-017-0351-z

**Published:** 2017-07-28

**Authors:** Martijn van Ee, René Sitters

**Affiliations:** 1 Vrije Universiteit Amsterdam, De Boelelaan 1105, 1081 HV Amsterdam, Netherlands; 2 Centrum voor Wiskunde en Informatica (CWI), Science Park 123, 1098 XG Amsterdam, Netherlands

**Keywords:** A priori optimization, Approximation algorithms, Traveling repairman problem

## Abstract

The field of a priori optimization is an interesting subfield of stochastic combinatorial optimization that is well suited for routing problems. In this setting, there is a probability distribution over active sets, vertices that have to be visited. For a fixed tour, the solution on an active set is obtained by restricting the solution on the active set. In the well-studied a priori traveling salesman problem, the goal is to find a tour that minimizes the expected length. In the a priori traveling repairman problem (TRP), the goal is to find a tour that minimizes the expected sum of latencies. In this paper, we study the uniform model, where a vertex is in the active set with probability *p* independently of the other vertices, and give the first constant-factor approximation for a priori TRP.

## Introduction

In the last few decades, a lot of research has been done in stochastic combinatorial optimization. This field is concerned with classical combinatorial optimization problems, like the shortest path problem and the minimum Steiner Tree problem, but with additional uncertainty in the instance. For example, there are situations where the problem instance changes on a daily basis. Instead of reoptimizing every instance, because it might be impossible or undesirable, one can alternatively choose to pick one solution that will be good on average. This is the setting of a priori optimization. In this paper, we consider the a priori traveling repairman problem (TRP). This is a routing problem, where there is a probability distribution over subsets of the vertices that have to be visited. A preliminary version of this paper was published in [19].

In a priori routing, we are given a complete weighted graph $G=(V,E)$ and a probability distribution on subsets of *V*. Depending on the model, this distribution is given either explicitly or by a sampling oracle. It is assumed that the instances are metric. In the first stage, a tour $\tau$ on *V* has to be constructed. In the second stage, an active set $A\subseteq V$ is revealed, which is the set of vertices to be visited. The second-stage tour $\tau _A$ is obtained by shortcutting the first-stage tour over the active set. For each active set, the first-stage tour has a second-stage objective value. The goal is to find a first-stage tour that minimizes the expected cost of the second stage tour. When it is clear form the context, we may refer to this expected second-stage cost simply as the expected cost of the solution.

In the literature, several models for the probability distribution over the active sets are used. In the *black-box* model [14], there is no knowledge on the probability distribution. The only instrument available is a sampling oracle, which gives a sample from the distribution on request. In the *scenario* model [11], the instance contains an explicit list with active sets and their corresponding probabilities. In the *independent decision* model [15], each vertex has its own probability of being active, independent of the other vertices. The special case where all probabilities are equal, i.e. $p_i=p$ for all *i*, is called the *uniform* model.

In the a priori traveling salesman problem (TSP), the goal is to minimize the expected length of the tour. The problem was introduced in the PhD-theses of Jaillet [12] and Bertsimas [2]. An approximation algorithm was achieved by Schalekamp and Shmoys [14], who showed that there is a $O({\log n})$-approximation algorithm in the black-box model. Later, Gorodezky et al. [10] showed that this bound is tight. Constant-factor approximations were achieved for the first time by Shmoys and Talwar [15], who showed that there exists a randomized 4-approximation and a deterministic 8-approximation in the independent decision model. The deterministic approximation guarantee was later improved to 6.5 by van Zuylen [20]. It is easy to show that the randomized 4-approximation can be improved to a factor $\alpha +2$ by replacing the double-tree subroutine in the algorithm of Shmoys and Talwar by an $\alpha$-approximation algorithm for TSP. Hence, using Christofides’ algorithm [5] gives a randomized 3.5-approximation. Note that the authors of [7] independently obtained a constant-factor approximation for a priori TSP in the independent decision model.

This paper is concerned with the a priori traveling repairman problem. In the deterministic traveling repairman problem or minimum latency problem, we have a complete graph $G=(V,E)$, a metric cost function *c* over the edges and a root vertex *r*. We want to find a tour $\tau$ starting at the root which minimizes the sum of latencies. Here, the latency of a vertex *v* is defined as the length of the path from *r* to *v* along $\tau$. The problem is known to be NP-hard in general [13] and it is even NP-hard on weighted trees [16]. The best known approximation guarantees are 3.59 for general metrics [4] and a polynomial time approximation scheme for the Euclidean plane and weighted trees [17]. The a priori traveling repairman problem is defined similarly to the a priori traveling salesman problem. The goal is to find a first-stage tour which minimizes the expected second-stage sum of latencies. Here, the second-stage sum of latencies for active set *A* is obtained by shortcutting the first-stage tour over *A* and summing up the latencies in the second-stage tour. In this paper, we establish a constant-factor approximation for the a priori traveling repairman problem in the uniform model. To achieve this result, we consider the a priori *k*-TSP, the prize-collecting tour single-sink rent-or-buy problem, and the a priori prize-collecting traveling salesman problem. These problems will be defined in their corresponding sections.

In the next section, the basic ideas for our algorithm for the a priori traveling repairman will be discussed. After that, it will be shown how the a priori *k*-TSP can be used to obtain a constant-factor approximation for a priori TRP on trees. In Sect. 5, we will discuss how to get a constant-factor approximation for the a priori TRP on general metrics. In order to get there, we investigate the tour single-sink rent-or-buy problem and its prize-collecting version. Finally, we end with some remarks on open problems.

In this paper, it is assumed that the edge costs are non-negative integers satisfying the triangle inequality. In the following, we denote an active set of vertices by *A*. When the set is drawn from a probability distribution, we denote the expectation with respect to this distribution as ${\mathbb {E}}_A[\cdot ]$.

## Preliminaries

In the decision version of the a priori traveling repairman problem in the independent decision model, we are given a weighted graph *G* with *n* vertices and root vertex *r*, probabilities $p_i$ for $i=1,\ldots ,n$ and a number *k*. Vertex *i* is active with probability $p_i$. Further, assume that the edge weights are rationals and that the smallest weight is equal to 1. The question is whether there exists a tour, starting at the root, that has an expected sum of latencies of at most *k*. The next theorem shows that this decision version is contained in NP. Since it generalizes TRP, the decision problem is NP-complete.

### Theorem 1

The decision version of a priori TRP in the independent decision model is in NP.

### Proof

Given a tour $\tau$, w.l.o.g. $\tau =(1,2,\ldots ,n)$, the contribution of edge (*i*, *j*) with $i<j$ is equal to

1 $$\begin{aligned} c_{ij}p_ip_j\prod _{k=i+1}^{j-1}(1-p_k)\sum _{k=0}^n {\mathbb {P}}({ Succ}(j)=k)(k+1), \end{aligned}$$

where ${\mathbb {P}}({ Succ}(j)=k)$ is the probability that exactly *k* vertices after *j* on $\tau$ are active. If we can compute these probabilities in polynomial time, then we can compute (1) in polynomial time for every edge and sum over all edges. To compute the previously mentioned probability for a given *j*, we define sets $S_t=[t]\setminus [j]$ for $j\le t\le n$. Let ${\mathbb {P}}(S_t,k)$ be the probability that there are exactly *k* active vertices in set $S_t$. In the end, we want to know ${\mathbb {P}}(S_n,k)={\mathbb {P}}({ Succ}(j)=k)$ for all $0\le k\le |S_n|$. Initially, we have the following probabilities.

$$\begin{aligned} {\mathbb {P}}(S_j,0)=1,\quad {\mathbb {P}}(S_j,1)=0,\\ {\mathbb {P}}(S_t,-1)=0,\quad t=j,\ldots ,n. \end{aligned}$$

We can now recursively find all probabilities by using that the following relation holds for $t=j+1,\ldots ,n$ and for $k=0,\ldots ,|S_t|$.

$$\begin{aligned} {\mathbb {P}}(S_t,k)=p_t{\mathbb {P}}(S_{t-1},k-1)+(1-p_t){\mathbb {P}}(S_{t-1},k). \end{aligned}$$

Note that the procedure above runs in polynomial time. The theorem follows. $\square$

The decision version of a priori TRP is also in NP in the scenario model. Since the input contains an explicit list of the scenarios, the second-stage latencies can simply be computed for each scenario.

**Fig. 1:**
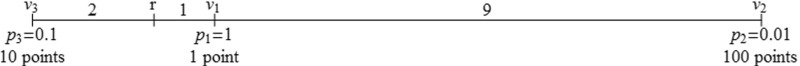
Instance of a priori TRP in the scenario model. The optimal tour passes the point at $v_1$ twice before visiting it

There are some intriguing difficulties with a priori TRP. Finding an approximation algorithm for this problem turns out to be much harder than for a priori TSP. It is easy to adjust the proof in [10] to show a $\Omega (\log n)$ lower bound on the approximation guarantee in the black-box model. Getting positive results is even non-trivial if all vertices are on a line. In the deterministic setting, TRP on the line can be solved using dynamic programming [1]. This result relies on the fact that vertices will always be visited when the tour comes across them. In the a priori setting, this is not true. Consider the example from the scenario model shown in Fig. 1.

### Example 1

There is a point at $v_1$ at distance 1 from the root which is always active. Further, there are 100 points at $v_2$ at distance 10 from the root which are simultaneously active with probability 0.01, and there are 10 points at $v_3$ at distance 2 on the other side of the root which are simultaneously active with probability 0.1. Note that this gives four possible scenarios. It is easy to compute that the optimal a priori tour is $(v_2,v_3,v_1)$, meaning that we pass by the point at $v_1$ twice before visiting it. The intuition behind this is that we do not want to visit $v_1$ before $v_3$, but we do want to visit $v_2$ before $v_3$. Hence, skipping may be optimal in the scenario model. However, we conjecture that in the independent model skipping is never optimal. If this is true, then dynamic programming may be used to solve this problem.

For general metric spaces, the independent decision model is non-trivial. The intuitive approach of using the probabilities as weights, i.e. $w_i=p_i$, and solving the weighted version of TRP turns out to give arbitrary bad solutions, as shown in Example 2.

### Example 2

Consider a star graph with $k+1$ leafs. Replace the last leaf with a clique containing $\ell$ vertices, with all edge weights equal to zero. Assign a weight of $\ell$ to the edge going to leaf $k+1$, and assign weight 1 to the remaining edges. Each vertex is active with probability $p=1/\ell$.

Now, if we take the probabilities as weights, we see that every solution (visiting all vertices of leaf $k+1$ at the same time) for the created weighted-TSP instance has the same value. However, in the a priori setting, it is optimal to visit leaf $k+1$ as last. Moreover, by choosing *k* properly and $\ell$ big, we can show that the ratio between the solution starting with leaf $k+1$ and the optimal solution is arbitrarily large.

On the other hand, the problem remains easy on star graphs. It can be shown by an interchange argument that the vertices have to be visited in non-increasing order of ${\mathbb {E}}[N_i]/{\mathbb {E}}[L_i]$. Here, ${\mathbb {E}}[N_i]$ is the expected number of clients at vertex *i* and ${\mathbb {E}}[L_i]$ is the expected length to vertex *i*, i.e. the length of the edge times the probability that at least one of the clients at the endpoint has to be visited. Even for slightly more general graphs, such as spiders of depth two, the complexity is still open.

## Algorithm

Before presenting our algorithm, we are going to rewrite the objective function and state a basic lemma that we will need in the analysis. Any tour should start in the given root *r*. For a given tour and active set *A*, we denote $\ell _i^A$ as the latency of vertex $i\in A$ in the tour shortcutted over *A*. If vertex *i* is not in *A*, then we define $\ell _i^A=0$. Each vertex *i* has probability $p_i$ of being active. If $C_i$ is the expected latency of vertex *i* given that *i* is active, the law of total probability gives that our objective becomes minimizing

2 $$\begin{aligned} {\mathbb {E}}_A\left[ \sum _{i}{\ell _i^A}\right] =\sum _{i}p_i{\mathbb {E}}_A\left[ \ell _i^A|i \text { is active}\right] =\sum _i p_i C_i. \end{aligned}$$

Let *d*(*r*, *i*) be the minimum cost of traveling from the root to vertex *i*. Note that $C_i$ is the expected latency of vertex *i*, *given* that it is active. Hence, we obtain the following lemma.

### Lemma 1

For any tour and vertex *i*, we have $C_i\ge d(r,i)$.

Our algorithm is based on algorithms for the deterministic TRP [3, 4, 8]. However, the a priori setting makes the problem a lot harder to solve. As explained above, even the problem on the line is non-trivial in the a priori setting and is not known to be solvable in polynomial time. Our algorithm makes use of an $(\alpha ,\beta )$-TSP-approximator in the a priori setting, which is similar to the one introduced in [3]. Suppose we have an instance of a priori TSP and a number *L*. The goal is to find a tour of expected length at most *L* which minimizes the number of unvisited vertices. An $(\alpha ,\beta )$-TSP-approximator in the a priori setting will find a tour of expected length at most $\beta L$ with a number of unvisited vertices at most $\alpha$ times the optimal number of unvisited vertices. More formally, it is defined as follows.

### Definition 1

An $(\alpha ,\beta )$-TSP-approximator in the a priori setting will find, for any given *L*, a tour that visits at least $(1-\alpha \epsilon )n$ vertices and is of expected length at most $\beta L$ if there exists a tour that visits $(1-\epsilon )n$ vertices and is of expected length *L*.

The algorithm works as follows. Let $L_0=2$ (twice the minimum edge length) and $c>1$ be a parameter to be determined later and define $L_i=L_0c^i$. Now for each length $L_i$, we obtain a tour $T(L_i)$ by applying the $(\alpha ,\beta )$-TSP-approximator in the a priori setting. These tours will then be concatenated, i.e. we first traverse tour $T(L_0)$, then we traverse tour $T(L_1)$ and so on until all vertices are visited, where we shortcut already visited vertices. We output the resulting tour.

### Theorem 2

Given an $(\alpha ,\beta )$-TSP-approximator in the a priori setting, our algorithm with $c=2$ is a $(8\lceil \alpha \rceil \beta +1)$-approximation for the a priori traveling repairman problem in the uniform model, i.e. $p_i=p$ for all $i\in V$.

### Proof

Assume that $\alpha$ is an integer, otherwise use its ceiling as upper bound. Partition the vertices of the *algorithm’s* tour in blocks of size at most $\alpha$. If we renumber the vertices in the tour such that the we have $(1,2,\ldots ,n)$, we define the block $B_x$ to be the subset containing the vertices $n-\alpha (x+1)+1,n-\alpha (x+1)+2,\ldots ,n-\alpha x$ for $x=0,1,\ldots ,\left\lceil \frac{n}{\alpha }\right\rceil -1$. Let $C_{n-x}^*$ denote the expected latency of vertex $n-x$, the $(n-x)$th vertex on the *optimal* a priori TRP-tour, given that it is active. Now let $S_i$ be the set of vertices with a conditional expected latency from $L_{i-1}$ until $L_i$ in the optimal tour. Suppose that the $(n-x)$th vertex visited by the optimal tour is in $S_i$, i.e. $L_{i-1}\le C_{n-x}^*<L_i$. We know that there exists a tour visiting at least $n-x$ vertices with expected length at most $2C_{n-x}^*\le 2L_i=L_{i+1}$, so the $(\alpha ,\beta )$-TSP-approximator (with respect to $L_{i+1}$) finds a tour visiting at least $n-\alpha x$ vertices of expected length at most $\beta L_{i+1}$. This implies that each vertex $v\in B_x$ is visited in $T_0\cup \cdots \cup T(L_{i+1})$. We can bound the conditional expected latency, denoted as $C_v^\text {Alg}$, in the following way. Let *v* be visited for the first time in $T(L_{i+1})$. Now, construct a new tour by removing vertex *v* from tour $T(L_{i+1})$ and visit it after the vertices of $T(L_{i+1})$. Denote the expected latency of *v* in the new tour by $C_v'$ and note that we have $C_v^\text {Alg}\le C_v'$. Finally note that the expected latency in the new tour is bounded by $\beta (L_0+\cdots +L_{i+1})+d(r,v)$. If we sum over all vertices in $B_x$, we get

$$\begin{aligned} \sum _{v\in B_x}C_v^\text {Alg}&\le \alpha (\beta (L_0+L_1+\cdots +L_{i+1})) +\sum _{v\in B_x}d(r,v)\\&\le 2\alpha \beta L_{i+1}+\sum _{v\in B_x}d(r,v)\\&= 8\alpha \beta L_{i-1}+\sum _{v\in B_x}d(r,v)\\&\le 8\alpha \beta C_{n-x}^*+\sum _{v\in B_x}d(r,v). \end{aligned}$$

If we multiply by *p* and sum over all blocks, we can bound the objective (2) as follows

$$\begin{aligned} \sum _{x=0}^{\left\lceil \frac{n}{\alpha }\right\rceil -1}\sum _{v\in B_x}p C_v^\text {Alg}&\le 8\alpha \beta \sum _{x=0}^{\left\lceil \frac{n}{\alpha }\right\rceil -1}p C_{n-x}^*+\sum _{v}p d(r,v)\\&\le 8\alpha \beta \sum _{v} p C_v^*+\sum _{v}p d(r,v)\\&\le (8\alpha \beta +1)\textsc {Opt}. \end{aligned}$$

$$\square $$

Note that uniformity is essential in the last step, since we are comparing different tours vertex by vertex. This approximation guarantee might be improved by choosing another value of *c*, but it turns out that $c=2$ is optimal for our analysis. We can improve the approximation factor by randomizing the starting length. Set $L_0=2c^U$, where *U* is a random variable uniformly distributed on [0, 1], and optimize over *c*.

### Theorem 3

Given an $(\alpha ,\beta )$-TSP-approximator in the a priori setting, our algorithm with $L_0=2c^U$ and $c=\mathrm {e}$ is a $(2\mathrm {e}\lceil \alpha \rceil \beta +1)$-approximation for the a priori traveling repairman problem in the uniform model, where *U* is a random variable uniformly distributed on [0, 1].

### Proof

Partition the vertices of the resulting tour in blocks of size at most $\alpha$ and renumber vertices as in Theorem 2. Suppose that $C_{n-x}^*=qc^\ell$, where $q<c$. If $q<c^U$, then there exists a path from the root with expected length at most $c^Uc^\ell$ visiting at least $n-x$ vertices. This means that $T(L_\ell )$ contains at least $n-\alpha x$ vertices and is of length at most $2\beta c^Uc^\ell$. So, for $v\in B_x$, we have $C_v^\text {Alg}\le \beta \sum _{i=0}^{\ell }L_0c^i+d(r,v)\le \beta L_0c^\ell (\frac{c}{c-1})+d(r,v)$. In the other case, we have $q<c\le c^Uc$, so there exists a path from the root with expected length at most $c^Uc^{\ell +1}$. This means that $T(L_{\ell +1})$ contains at least $n-\alpha x$ vertices and is of length at most $2\beta c^Uc^{\ell +1}$. So, for $v\in B_x$, we have $C_v^\text {Alg}\le \beta \sum _{i=1}^{\ell +1}L_0c^i+d(r,v)\le \beta L_0c^{\ell +1}(\frac{c}{c-1})+d(r,v)$. In the first case, we have $\log _c{q}\le U\le 1$ and we have $0\le U\le \log _c{q}$ in the second case. Taking expectations over *U* gives

$$\begin{aligned} C_v^\text {Alg}\le&\int _{\log _c{q}}^{1}\left( {\beta L_0c^\ell \left( \frac{c}{c-1}\right) +d(r,v)}\right) {\mathrm {d}}U\\&+\int _{0}^{\log _c{q}}\left( {\beta L_0c^{\ell +1}\left( \frac{c}{c-1}\right) +d(r,v)}\right) {\mathrm {d}}U\\ =&\frac{2c\beta }{\ln {c}}C_{n-x}^*+d(r,v) \end{aligned}$$

If we multiply by *p* and sum over all vertices in $B_x$ and over all $B_x$, we get a bound of $\frac{2c}{\ln {c}}\alpha \beta +1$. Optimizing over *c* gives $c=\mathrm {e}$ and a bound of $2\mathrm {e}\alpha \beta +1$. $\square$

The algorithm can be derandomized by trying multiple values for *U*. This will give an approximation guarantee that is arbitrary close to $2\mathrm {e}\alpha \beta +1$ by using techniques from [8]. Note that if $\alpha =1$, the approximator corresponds to a $\beta$-approximation for a priori *k*-TSP, the problem of finding a tour on *k* vertices of minimum expected length. This yields the following corollary.

### Corollary 1

If there is a $\gamma$-approximation for the a priori *k*-TSP, then there is a $(2\mathrm {e}\gamma +1)$-approximation for the a priori traveling repairman problem in the uniform model.

## Tree Metrics

To obtain an approximation guarantee for the a priori TRP on trees, we use Corollary 1. Note that finding a *k*-tour in a tree is similar to finding a *k*-tree in a tree. So, in this case we can solve the a priori *k*-MST problem, in which we have to find a tree spanning *k* vertices such that the expected cost of the tree is minimized. Here, shortcutting the tree is done by removing inactive vertices provided that the tree on the active vertices remains connected.

### Theorem 4

The a priori *k*-TSP in the uniform model on tree metrics can be solved to optimality in polynomial time.

### Proof

First, we turn the tree into a binary tree with the original vertices at the leaves by adding vertices with probability zero and edges with cost zero. Next, we use dynamic programming to solve the a priori *k*-MST problem. Define the function *t*(*v*, *y*) to be the minimal expected cost of a subtree rooted at *v* containing *y* leaves. For all leaves *v*, we have $t(v,0)=t(v,1)=0$. For a certain state (*v*, *y*), the best tree follows from a combination of *z* vertices from the left subtree and $y-z$ vertices from the right subtree. For a given combination, the expected cost is equal to the sum of the expected cost of the subtrees plus, for each subtree, the cost of the edge connecting *v* with the subtree times the probability that at least one of the vertices in the subtree is active. If we denote $\ell (v)$ and *q*(*v*) for the left and right child of *v* respectively and *c*(*v*, *w*) as the cost of the edge between *v* and *w*, we get the following recursive formula:

$$\begin{aligned} t(v,y)=&\min _{z=0,\ldots ,y}\Big \{t(\ell (v),z)+(1-(1-p)^z)c(v,\ell (v))\\&+t(q(v),y-z)+(1-(1-p)^{y-z})c(v,q(v))\Big \}. \end{aligned}$$

The optimal tree containing *k* vertices is the solution corresponding to *t*(*r*, *k*), where *r* is the root of the tree. Note that the dynamic program needs $O(nk^2)$ time, so a priori *k*-MST (and hence *k*-TSP) on trees can be solved in polynomial time. $\square$

### Corollary 2

There is a $2\mathrm {e}+1\approx 6.44$-approximation for the a priori traveling repairman problem in the uniform model on trees.

It is not clear how to generalize this result to the non-uniform case. The difficulty is that the probability that at least one vertex in the subtree is active can take exponentially many different values. On the other hand, it is easy to extend the DP above to the case where it is almost uniform in the sense that there is a constant number of different probabilities $p_i$.

## General Metrics

For general metrics, we show how to obtain an $(\alpha ,\beta )$-TSP-approximator with some constant $\alpha$ and $\beta$. It turns out that finding such an approximator boils down to finding an approximation algorithm for certain variations of the tour single-sink rent-or-buy problem (tour SRoB).

In the single-sink rent-or-buy problem (SRoB) [18], we are given a graph $G=(V,E)$ with a metric cost function $c_e$ on the edges. There is a client at every vertex $j\in V$ with demand $d_j$. We have to open a facility at some of the vertices and connect the clients to the facilities. We denote $c_{ij}$ as the cost of the shortest path between *i* and *j* in *G*. Connecting facility *i* with client *j* costs $d_jc_{ij}$ and buying edge *e* costs $Mc_e$, where $M\ge 1$. We need to buy edges such that the open facilities are joined by a Steiner tree, where the open facilities are the terminals. The goal is to minimize the sum of connection cost and Steiner cost.

In the tour SRoB, *G* is a complete graph. Here, edges have to be bought such that the open facilities are joined by a tour. Note that $c_{ij}=c_e$ if $e=(i,j)$.

The next two variants are used to get the desired approximation results for a priori TRP. In the prize-collecting tour SRoB, it is not needed to connect every client, but if client *i* is not connected, then we have to pay penalty $\pi _i$. The goal is to minimize the sum of connection cost, tour cost and penalty cost.

In the *k*-client tour SRoB, it also not needed to connect every client. One has to connect at least *k* vertices at minimum total cost. Approximating the latter problem is done by using the following definition.

### Definition 2

An $(\alpha ,\beta )$-tour SRoB-approximator will find, for any given *L*, a tour SRoB-solution containing at least $(1-\alpha \epsilon )n$ vertices of cost at most $\beta L$ if there exists a tour SRoB-solution containing $(1-\epsilon )n$ vertices of cost *L*.

In this section, we start with showing that there is a 5-approximation for tour-SRoB. We then use this result to show that there is 5.52-approximation for the prize-collecting tour SRoB. In Sect. 5.2, we first show that if we have an $(\alpha ,\beta )$-tour SRoB-approximator, we get an $(\alpha ,3\beta )$-TSP-approximator. Finally, we show that the 5.52-approximation for prize-collecting tour SRoB can be used to obtain an (11.04, 11.04)-tour SRoB-approximation which together with the former statement results in an (11.04, 33.12)-TSP-approximator. Hence, by Theorem 3 this results in a *O*(1)-approximation for a priori TRP in the uniform model on general metrics.

### Prize-Collecting Tour SRoB

The prize-collecting tour SRoB has, to the best of our knowledge, not been considered explicitly in the literature. We can obtain a randomized 3-approximation for tour SRoB by adjusting the analysis for tour connected facility location (a generalization of tour SRoB) by Eisenbrand et al. [6]. This can be derandomized by adapting the analysis of van Zuylen [20] to obtain a deterministic 3-approximation. However, it is not clear how to extend these results to prize-collecting SRoB. Therefore, we will use the primal-dual algorithm for SRoB by Swamy and Kumar [18] instead.

#### Tour SRoB

First, consider SRoB. We assume that a facility is opened at root vertex *r*. In the ILP-formulation below, we define $x_{ij}$ to be 1 if *i* is on the tree and *j* is connected to *i*. We define $z_e$ to be 1 if we use edge *e* in the tree. Without loss of generality, we assume that we have unit demand. The reader is referred to [18] for further details.Relaxing the integrality constraints gives the following dual problem.In any solution for the tour SRoB, each vertex *j* is connected to some vertex *i* on the tour (possibly $i=j$). In that case, any cut separating *i* from *r* must contain at least two edges. Hence, an LP-relaxation for tour SRoB is obtained by relaxing the integrality constraints in (P) and by putting a factor 2 in front of $x_{ij}$ in the second constraint. We obtain the following LP-relaxation and its dual.
We can now use the primal-dual algorithm for SRoB to obtain an approximation algorithm for tour SRoB. Given an instance of tour SRoB, we divide all edge costs by 2, i.e. $c_e'=c_e/2$ and $c_{ij}'=c_{ij}/2$. To keep the remaining restrictions of the dual and the Steiner costs the same, we also set $M'=2M$. Secondly, we use the primal-dual algorithm of Swamy and Kumar [18] on the new instance to obtain a solution for SRoB. Finally, we double the tree and shortcut the resulting Eulerian tour. Note that this algorithm and its analysis are similar to the work of Goemans and Williamson [9], who showed how to obtain a 2-approximation for the prize-collecting TSP using a 2-approximation for the prize-collecting Steiner tree problem. Further note that this ratio is worse than the ratio that can be obtained from [6]. However, that result is based on a sampling approach which we do not know how to extend to the prize-collecting version of the problem.

##### Theorem 5

The approach above gives a 5-approximation for the tour SRoB. Moreover, the value is at most 5 times the optimal value of its LP-relaxation.

##### Proof

The primal-dual algorithm of Swamy and Kumar gives two feasible solutions, namely $(\alpha ^1,\theta ^1)$ and $(\alpha ^2,\theta ^2)$. Then, $(2\alpha ^1,\theta ^1)$ and $(2\alpha ^2,\theta ^2)$ are feasible solutions for $\text {D}'$. By duality, we have $2\sum _j\alpha _j^1\le \textsc {Opt}$ and $2\sum _j\alpha _j^2\le \textsc {Opt}$, where $\textsc {Opt}$ is the optimal value for tour SRoB. Given the solution of SRoB with connection costs $C'$ and Steiner cost *S*, the cost of the solution for tour SRoB produced by the algorithm is at most $C+2S=2(C'+S)$. By Swamy and Kumar, we get $C'+S\le 3\sum _j\alpha _j^1+2\sum _j\alpha _j^2$. Combining these two equations, we get that the solution of our algorithm has cost at most

$$\begin{aligned} C+2S = 2(C'+S) \le 2\left( 3\sum _j\alpha _j^1+2\sum _j\alpha _j^2\right) \le 3\textsc {Opt}+2\textsc {Opt}\le 5\textsc {Opt}. \end{aligned}$$

Note that the solution of our algorithm contains a tour on the open facilities and it is therefore a feasible solution for tour SRoB. $\square$

#### The Prize-Collecting Version

In this version, it is not needed to connect all vertices. However, a penalty $\pi _i$ is incurred when vertex *i* is not connected. For the LP-relaxation of the prize-collecting tour SRoB problem, we add the variable $s_j$, which is set to 1 if client *j* is not connected. In an integral solution, the first constraint corresponds to a client being either connected with an open facility or not connected at all.Using the ellipsoid method, the LP-relaxation can be solved in polynomial time. Note that the separation problem can be solved by using a min-cut algorithm. The algorithm for the prize-collecting version works as follows (see [21], Sect. 4.4). Let $(x^*,z^*,s^*)$ be an optimal solution for $(\text {P}'')$. If $s_j^*\ge \delta$, then we set $\hat{s_j}=1$, else we set $\hat{s_j}=0$, where $0\le \delta \le 1$ is determined later, and let $T=\{j:\hat{s}_j=0\}$. The vertices in $V\setminus T$ will not be visited. Next, we obtain a solution of tour SRoB on *T* by applying the algorithm from Theorem 5. This results in a feasible solution for prize-collecting tour SRoB on *V*. Partition the optimal LP-value in the connection plus tour cost $C_{\text {LP}}$ and penalty cost $\varPi _{\text {LP}}$.

##### Lemma 2

The algorithm above finds a solution for the prize-collecting tour SRoB such that the resulting tour and connection cost is bounded by $5/(1-\delta )C_{\text {LP}}$ and the resulting penalty cost is bounded by $(1/\delta )\varPi _{\text {LP}}$.

##### Proof

By rounding the solution, we lose at most a factor $1/\delta$ on the penalty cost. This means that the penalty cost is at most a factor $1/\delta$ times the penalty cost of the LP-relaxation. By Theorem 5, the connection and tour cost for tour SRoB on *T* can be bounded by 5 times the optimal solution of its LP-relaxation. We obtain a feasible solution for this LP-relaxation by deleting the $s_j$’s from the LP-relaxation of prize-collecting tour SRoB and multiply all other variables by a factor $1/(1-\delta )$. Combining the two statements, we obtain that the connection and tour cost can be bounded by $5/(1-\delta )$ times the connection and tour cost of the optimal LP-solution. $\square$

If we choose $\delta$ uniformly at random on $\left[ 0,\theta \right]$, with $0<\theta \le 1$ to be specified later (see [21], Sect. 5.7), we obtain the following result.

##### Lemma 3

Randomization of the algorithm above gives a solution for the prize-collecting tour SRoB such that the resulting tour plus connection cost is in expectation bounded by $(5\ln \left( 1/(1-\theta )\right) /\theta ) C_{\text {LP}}$ and the resulting penalty cost is in expectation bounded by $(1/\theta )\varPi _{\text {LP}}$.

##### Proof

The tour and connection costs are deterministically bounded by $5/(1-\delta )C_{\text {LP}}$. If we take the expected value with respect to $\delta$, we get that the tour and connection costs are bounded by ${\mathbb {E}}\left( 5/(1-\delta )\right) C_{\text {LP}}$ in expectation. Computing this expectation gives:

$$\begin{aligned} {\mathbb {E}}\left( \frac{5}{1-\delta }\right) =\int _{0}^{\theta }\frac{5}{1-x} \frac{1}{\theta }{\mathrm {d}}x=-\left. \frac{5}{\theta }\ln {(1-x)}\right| _{0}^\theta =\frac{5}{\theta }\ln \left( \frac{1}{1-\theta }\right) . \end{aligned}$$

If $s_j^*<\theta$, then $s_j^*$ gets rounded to 1 (i.e. *j* will not be visited) with probability $s_j^*/\theta$. If $s_j^*\ge \theta$, then $s_j^*$ gets rounded to 1 with probability 1, but here we have $s_j^*/\theta \ge 1$. So, we can bound the penalty cost by $(1/\theta )\sum _j s_j^*\pi _j\le (1/\theta ) \varPi _{\text {LP}}$. $\square$

Note that the algorithm can be derandomized by checking all values $s_j^*\in \left[ 0,\theta \right]$ for $\delta$, since the set of unvisited vertices does not change for values in between two consecutive values of $s_j^*$. So, by checking at most *n* values, we obtain a deterministic algorithm with the same guarantees. Choosing $\theta =1-\mathrm {e}^{-1/5}$ gives the following approximation guarantee.

##### Theorem 6

There is a 5.52-approximation for the prize-collecting tour SRoB problem.

### Obtaining an $(\alpha ,\beta )$-TSP-Approximator

In this subsection, it is shown how to obtain an $(\alpha ,\beta )$-TSP-approximator using the results for prize-collecting tour SRoB. We first show how a priori TSP and tour SRoB are related.

#### Lemma 4

Any approximation algorithm for the tour SRoB problem can be turned into an approximation algorithm for the a priori TSP in the independent decision model with loss of at most a factor 3 in the approximation.

#### Proof

Given an instance of a priori TSP with edge costs $c_e$ and probabilities $p_i$, we define an instance of tour SRoB as follows. The edge costs are $c_e'=c_e$ $\forall e$, $M=1$ and the demands are $d_i=2p_i$. Given any feasible solution for this instance we get a feasible solution for a priori TSP of at most the same cost as follows. Let *T* be the tour in the SRoB solution. For the a priori tour we take *T* and double all the edges from clients to facilities in the SRoB solution. It is easy to see that the expected cost of the shortcut TSP solution is at most that of the SRoB solution. Let $\textsc {Opt}_{\text {TSP}}$ and $\textsc {Opt}_{\text {SRoB}}$ denote the optimal value of, respectively, the a priori TSP and the tour SRoB instance. It remains to show that $\textsc {Opt}_{\text {SRoB}}\le 3 \textsc {Opt}_{\text {TSP}}$. Select vertex *i* with probability $p_i$ and take an optimal tour on the set of selected vertices *S*. Let this be the tour for the SRoB solution. Connect all other vertices in the cheapest way to *S*. It follows from the analysis in [15] that the cost of this SRoB solution is at most 3 times the optimal cost of the a priori TSP instance, since the construction above is just their algorithm except for the fact that we take an optimal tour on *S*. Hence, $\textsc {Opt}_{\text {SRoB}}\le 3 \textsc {Opt}_{\text {TSP}}$. $\square$

The theorem above applies as well in the *k*-client setting, both for regular $\alpha$-approximations and for $(\alpha ,\beta )$-tour SRoB-approximators.

#### Corollary 3

In the independent decision model, we have that

1. If there is an $\alpha$-approximation for the *k*-client tour SRoB problem, then there is a $3\alpha$-approximation for the a priori *k*-TSP.
2. If there is an $(\alpha ,\beta )$-tour SRoB-approximator, then there is an $(\alpha ,3\beta )$-TSP-approximator in the a priori setting.

#### Proof

In both cases, we use the same transformation as in the proof of Lemma 4.

1. The proof is working similarly, except that in the last step, we need to sample *S* from the vertices on the optimal *k*-tour, instead of sampling *S* from all vertices *V*. By Lemma 4, we get a $3\alpha$-approximation.
2. We sample *S* from the vertices on the optimal *k*-tour. Note that the number of visited vertices in the obtained a priori *k*-TSP solution is the same as in the optimal a priori *k*-TSP solution, so we do not lose anything there. Hence, by Lemma 4, we obtain an $(\alpha ,3\beta )$-TSP-approximator in the a priori setting. $\square$

Finally, we obtained the next lemma which shows that an $(\alpha ,\beta )$-tour SRoB-approximator can be obtained using results from prize-collecting tour SRoB.

#### Lemma 5

If there is an $\alpha$-approximation for prize-collecting tour SRoB, then there is a $(2\alpha ,2\alpha )$-tour SRoB-approximator.

#### Proof

Assume that there exists a solution *T* of expected cost at most *L* which visits at least $(1-\epsilon )n$ vertices. We show how to get a tour of expected cost at most $2\alpha L$ that visits at least $(1-2\alpha \epsilon )n$ vertices. As noted in [3], we can perform a binary search on the optimal value of $\epsilon$ given *L*, if $\epsilon$ is not specified. Define an instance of prize-collecting tour SRoB by giving each vertex a penalty $\pi =L/(\epsilon n)$. The optimal value of this instance is at most that of solution *T* which is $L+\epsilon n\pi \le 2L$. Hence, any $\alpha$-approximation for the prize-collecting tour SRoB instance should return a solution that has tour and connection cost at most $2\alpha L$ and also a penalty cost of at most $2\alpha L$. The latter implies that it leaves at most $2\alpha L/\pi =2\alpha \epsilon n$ vertices unvisited. $\square$

Now, we finally get a constant-factor approximation algorithm for the a priori TRP in the uniform setting.

#### Theorem 7

There is an *O*(1)-approximation for the a priori traveling repairman problem in the uniform model.

#### Proof

From Theorem 6 we get an $\alpha _0$-approximation for the prize-collecting tour SRoB, where $\alpha _0=5.52$. Combining this with Lemma 5, we obtain an $(2\alpha _0,2\alpha _0)$-tour SRoB-approximator. Using Corollary 3, we get an $(2\alpha _0,6\alpha _0)$-TSP-approximator. Plugging this results into the result of Theorem 3, we obtain a $(2\mathrm {e}\lceil 2\alpha _0 \rceil 6\alpha _0 +1)$-approximation for the a priori TRP in the uniform model. $\square$

## Open Problems

There are still many open problems in the field of a priori optimization. For the a priori traveling repairman problem we were only able to give a constant-factor approximation in the uniform model and the constant is still large. For the correctness of Theorems 2 and 3 the uniformity of the probabilities is essential. It is not clear how to reduce the case of independent probabilities to the uniform model. Therefore, the problem is wide open in the independent decision model with non-uniform probabilities. Also, it is not known if the uniform problem can be solved efficiently in case all points are on the line. If any optimal solution has the property that no point is passed without visiting it, like in the deterministic problem, then the problem may be solved by dynamic programming. However, a proof of this property is missing and we have shown that this property does not hold in the scenario setting.

In our analysis we used the theory of $(\alpha ,\beta )$-TSP-approximators. Better approximations may be obtained by using the a priori *k*-TSP or *k*-client tour SRoB. No constant-factor approximation is known for these problems.

Finally, it is good to note that there is still a lot to do in the scenario model. Both the a priori TSP and a priori TRP have not been studied in this model. It would be interesting to see if this extra knowledge, i.e. an explicit list of scenarios, can help us to obtain stronger approximation results.
